# Molecular Chaperones’ Potential against Defective Proteostasis of Amyotrophic Lateral Sclerosis

**DOI:** 10.3390/cells12091302

**Published:** 2023-05-02

**Authors:** Sumit Kinger, Ankur Rakesh Dubey, Prashant Kumar, Yuvraj Anandrao Jagtap, Akash Choudhary, Amit Kumar, Vijay Kumar Prajapati, Rohan Dhiman, Amit Mishra

**Affiliations:** 1 Cellular and Molecular Neurobiology Unit, Indian Institute of Technology Jodhpur, Jodhpur 342037, India; 2 Discipline of Biosciences and Biomedical Engineering, Indian Institute of Technology Indore, Simrol, Indore 453552, India; 3 Department of Biochemistry, School of Life Sciences, Central University of Rajasthan, Ajmer 305817, India; 4 Laboratory of Mycobacterial Immunology, Department of Life Science, National Institute of Technology, Rourkela 769008, India

**Keywords:** ALS, chaperones, HSP70, HSP27, HSPB8, HSP40

## Abstract

Amyotrophic lateral sclerosis (ALS) is a neuronal degenerative condition identified via a build-up of mutant aberrantly folded proteins. The native folding of polypeptides is mediated by molecular chaperones, preventing their pathogenic aggregation. The mutant protein expression in ALS is linked with the entrapment and depletion of chaperone capacity. The lack of a thorough understanding of chaperones’ involvement in ALS pathogenesis presents a significant challenge in its treatment. Here, we review how the accumulation of the ALS-linked mutant FUS, TDP-43, SOD1, and C9orf72 proteins damage cellular homeostasis mechanisms leading to neuronal loss. Further, we discuss how the HSP70 and DNAJ family co-chaperones can act as potential targets for reducing misfolded protein accumulation in ALS. Moreover, small HSPB1 and HSPB8 chaperones can facilitate neuroprotection and prevent stress-associated misfolded protein apoptosis. Designing therapeutic strategies by pharmacologically enhancing cellular chaperone capacity to reduce mutant protein proteotoxic effects on ALS pathomechanisms can be a considerable advancement. Chaperones, apart from directly interacting with misfolded proteins for protein quality control, can also filter their toxicity by initiating strong stress-response pathways, modulating transcriptional expression profiles, and promoting anti-apoptotic functions. Overall, these properties of chaperones make them an attractive target for gaining fundamental insights into misfolded protein disorders and designing more effective therapies against ALS.

## 1. Introduction

Neurodegenerative disorders represent a significant burden in aging populations globally. The pathogenesis of such diseases is often associated with the accumulation of mutant aberrantly folded proteins. Amyotrophic lateral sclerosis (ALS) is one such motor neuronal degenerative condition, often involving pathogenic neuronal hyper-excitability [1]. The primary phenotype of ALS involves debilitating muscular atrophy, which rapidly progresses from the period of clinical diagnosis. The disease is characterized by damages in motor neurons from the primary motor cortex and spinal cord along with abnormalities in the brainstem. Based on which types of motor neuronal cells are affected first, this disease can be classified into bulbar onset, affecting the muscles involved in speaking and swallowing, or spinal onset, affecting the skeletal muscles of limbs [2]. These muscular dysfunctions lead to a difficulty in performing simple daily life tasks and in advanced stages may hamper diaphragm functioning, leading to respiratory failure. The post-mortem studies on ALS patients’ neurons demonstrate the accumulation of mutant proteins. In both sporadic (sALS) and familial (fALS) cases, four major mutant proteins are reported to form toxic inclusions, namely, TAR DNA-binding protein 43 (TDP-43), superoxide dismutase 1 (SOD1), fused in sarcoma (FUS), and chromosome 9 open reading frame 72 (C9orf72) [3,4,5,6,7,8,9,10,11]. The ALS-linked variants can affect multiple cellular processes, including DNA repair, nuclear–cytoplasmic transport, mitochondrial function, endoplasmic reticulum (ER) homeostasis, vesicular transport, and synaptic functions, among others.

Multiple genetic mutations are associated with pathomechanisms of disease. The disease-associated mutations can be inherited from the family or acquired through sporadic mutational events. The sporadic mutations are much more prevalent in causing ALS compared to familial mutations. Several studies have found mutations in *SOD1*, *FUS*, and *TARDBP* to be more common in Asian populations, whereas *FUS* and *C9orf72* mutations are more common in Western countries [12,13,14]. Moreover, recent investigations have pointed out several other mutations associated with sALS, such as *OPTN* and *ATXN2* [15,16,17]. Due to such a complex genetic mutational basis, it is difficult to understand which of the mutant proteins formulate the underlying cause of ALS pathogenesis and how the occurrence of one mutant protein modulates that if another. Furthermore, the differences among ethnicities also affect the incidence of mutants linked with disease pathology. The mutations in C9orf72, TDP-43, SOD1, and FUS leading to a protein accumulation phenotype present in ALS are well characterized. Moreover, ALS is often found to have misfolded protein accumulations, which are believed to be central to its pathomechanism. Such a scenario encouraged us to review the mechanism of damages induced during the aberrant build-up of such mutant proteins in ALS. 

The proteins synthesized from *SOD1*, *FUS*, and *TARDBP* genes with ALS-associated point mutations and dipeptide repeats (DPRs) synthesized from the *C9orf72* gene with pathogenic nucleotide repeat expansion are prone to forming aggregates [18,19,20,21,22]. Such mutant proteins may accumulate and self-associate or interact with other cellular elements, generating toxic protein inclusions in ALS [23]. To counter the build-up of such toxic molecules and ensure their removal from the cytoplasm, cells employ a strict protein quality control (PQC) mechanism. The PQC machinery constitutes three central arms involving chaperones, the ubiquitin proteasome system (UPS), and autophagy [24]. Misfolded polypeptides are marked by a small ubiquitin protein for their clearance by proteolysis in the proteasome or lysosome. The addition of the ubiquitin chain is highly regulated and performed by the concerted action of E1, E2, and E3 enzymes [25]. Chaperones constitute the principal molecules of the cellular proteostasis network that maintains the conformational landscape of the proteins [26]. The primary regulation of misfolding events involves critical surveillance by chaperones initiating a triage between refolding and clearance. Failure of chaperones can, therefore, enhance the accumulation of toxic proteins, overburdening the cellular protein degradation machinery [27]. The motor neuron pathology in ALS is also reported to have reduced chaperone functioning, possibly leading to a decline in neuronal health [28]. Such reports present chaperones as modulators of neuronal degeneration conditions. Moreover, the implicated chaperones are found to specifically decrease the accumulation of major pathogenic ALS-linked proteins and reduce cellular toxicity by enhancing cytoprotective measures. Thus, we have attempted to represent the contribution of a few critical chaperones that are involved in ALS pathology and can potentially act as effective therapeutic targets. Furthermore, we have also highlighted key questions and challenges to be addressed in this domain and justified the therapeutic potential of chaperones.

## 2. The Cellular and Molecular Pathological Hallmarks of ALS

Misfolded protein accumulation disturbs cellular homeostasis, leading to molecular toxicity, involving a decline in PQC and aggravating cellular apoptosis. It is essential to gain deeper insight into how misfolded proteins damage the homeostatic pathways to identify critical targets for circumventing the activation of cellular death.

### 2.1. Loss of Genomic Damage Repair

**Figure 1:**
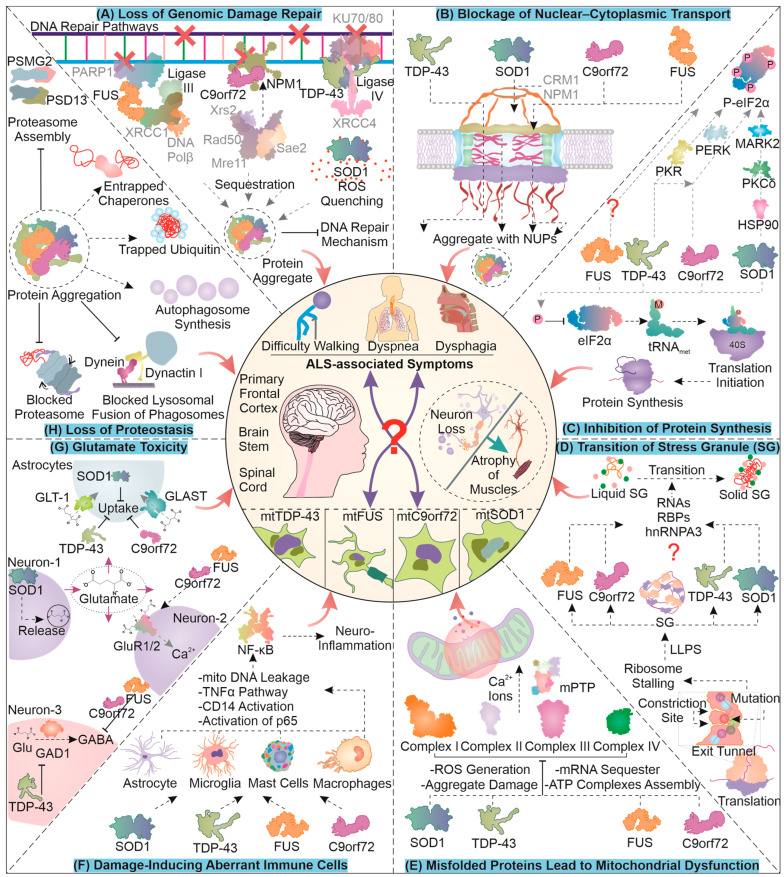
**The Cellular and Molecular Pathological Aspects of ALS:** A compilation of the mechanisms of cellular damage due to four major ALS-related mutant proteins; viz., TDP-43, FUS, SOD1, and C9orf72 are presented here. The type of cellular and molecular anomalies illustrated in the figure can formulate critical features of protein aggregation-based damages in neurodegenerative disorders such as ALS. For mechanistic details of individual damages, follow the description in the text.

The accumulation of mutant proteins can eventually enhance the formation of free radicals in cells generating single- and double-stranded DNA breaks. Furthermore, the mutation and aggregation of ALS-associated proteins can compromise genome damage repair mechanisms (Figure 1A). For instance, FUS is involved in DNA damage repair, and its mutation-based aggregation can restrict cellular potential to overcome nuclear damage [29]. Essentially, FUS is involved in recruiting XRCC1/DNA ligase IIIα with the help of PARP1 towards DNA damage sites, and upon its aggregation, DNA ligation is attenuated [30]. Similarly, TDP-43 is known to enhance the loading of XRCC4/DNA ligase IV at the site of damage in the NHEJ repair pathway, and upon its mutation, DNA repair is compromised [31,32]. C9orf72 DPRs are reported to alter the NHEJ as well as MMEJ DNA repair pathways by sequestering NPM1, which is an integral member of both [33]. Upon DNA damage, its sensors ATM/Chk2 and ATR/Chk1 can potentially phosphorylate SOD1, enhancing its nuclear localization to prevent further DNA oxidative damage. Contrarily, point mutant SOD1G93A is reported to inhibit the nuclear localization of wild-type SOD1, possibly contributing to enhanced oxidative DNA damage [34,35]. These reports demonstrate the critical implications of mutant ALS-related proteins for further enhancing DNA damage and possibly generating by-product mutants during ALS pathogenesis.

### 2.2. Blockage of Nuclear–Cytoplasmic Transport

Generally, the movement of proteins across the nucleus is highly regulated with the help of large protein complexes including the nuclear pore complex (NPC) and several accessory proteins. The dysregulation of nuclear–cytoplasmic transport is observed as a characteristic feature in ALS (Figure 1B). This dysregulation could, in part, be caused due to the co-localization of ALS-linked mutant proteins, such as TDP-43 and FUS, with NPC members, which may drive their aggregation [36,37]. In a SOD1 ALS mouse model, mislocalization of nucleoporins was observed, and in turn, these nucleoporins were co-localized with TDP-43, possibly contributing to pathogenesis [38]. An interesting study showed that mutation in SOD1 can expose its nuclear exit sequence (NES) and enhance the removal of mutant SOD1 from the nucleus with the help of nuclear export protein CRM1. This study also implies that such an egress of the mutant form of SOD1 from the nucleus could be a protective measure as the elimination of NES from mutant SOD1 enhanced toxicity and the associated symptoms in *C. elegans* [39]. Furthermore, another investigation conducted on mice showed the egression of mutant SOD1 from the nucleus, possibly with the help of the spinal motor neuron (SMN) protein complex [40]. An interesting consequence of nucleo–cytoplasmic transport proteins’ mislocalization or aggregation could possibly be the dysregulation of DNA repair; for instance, certain nucleoporins are involved in DNA repair [41]. 

Similarly, in the presence of C9orf72-based dipeptide repeats (DPRs) or its hexanucleotide repeat-expanded RNA, altered import and export across nucleus are observed, which may also be responsible for TDP-43 mislocalization in the cytoplasm [42,43,44,45]. Furthermore, nuclear–cytoplasmic transport defects caused by DPRs, arsenite, or sorbitol stress, have been attributed to the generation of stress granules, as they possibly sequester essential nuclear transport elements in them [46,47]. Interestingly, a few recent studies have shown that certain C9orf72 DPRs, as well as stress granules, might not necessarily lead to transport defects [48,49]. There is limited understanding of stress granules as a causative factor of nuclear transport defects. However, it is comparatively clear that the ALS pathology is associated with problems in the nuclear transport mechanism and the generation of stress granules.

### 2.3. Toxic Transition of Stress Granules

ALS-associated proteins may also lead to the liquid–liquid phase separation (LLPS)-dependent generation of stress granules (SGs). These granules are rich in stalled ribosomes, RNA, and several RNA-binding proteins. SGs are cytoplasmic structures, which upon interaction with mutant proteins can lose their dynamicity and form toxic non-dynamic (solid-like) granules. The mutant protein residues can assume different interactions with the ribosomal proteins in the exit tunnel during translation (Figure 1D). These altered interactions can help the ribosome to identify mutant proteins, and often the ribosome responds to this by stalling the translation process [50]. The accumulation of stalled ribosomes can lead to an increased interaction between proteins and RNA, leading to the synthesis of stress granules [51]. During the pathogenesis of ALS, SOD1G93A is known to localize with RNA and RNA-binding proteins involved in stress granule synthesis. This localization of mutant SOD1, with time, can generate non-dynamic areas of stress granules and form non-dissolving types of aggregates inside the granules, reducing their dynamic nature [52].

Moreover, mutant FUS can localize towards stress granules and also sequester hnRNPA3 with them, forming insoluble aggregates [53,54]. C9orf72-based DPRs can also co-accumulate with several RNA and RNA-binding molecules, initiating the association between the RNAs and proteins, forming liquid-like stress granules. These formations also undergo a transition to a solid-like state and limit the dynamicity of the granules implicated in cellular toxicity [55,56]. Furthermore, TDP-43 mutants can accumulate in SGs by interacting with proteins responsible for SG formation, such as TIA-1 [57]. Aberrant accumulations of mutant TDP-43 in the cell can reduce SG formation, due to the decreased levels of proteins required for SG synthesis [58]. Another report demonstrated that siRNA-dependent reduction of TDP-43 can lead to a loss of SG dynamicity, suggesting the significance of TDP-43 in regulating SG nature [59]. The synthesis of stress granules is initially thought to be cytoprotective in nature and adopted as a response to stress. Due to the co-accumulation of such mutant proteins, their clearance can be affected, leading to the generation of granules of a more solid nature.

### 2.4. Inhibition of Protein Synthesis

Stalling of ribosomes may also be initiated in response to various stress conditions in the cell that are sensed with the help of diverse stress sensors (Figure 1C). One of the prominent converging points of the stress signals, the integrated stress response (ISR), involves the phosphorylation of eIF2α (protein synthesis initiator). Four major kinases can mediate the phosphorylation of eIF2α, namely, PERK, GCN2, PKR, and HRI. Mutant TDP-43 hampers protein synthesis by instigating PERK branch-associated eIF2α phosphorylation [60]. Moreover, a knockdown of TDP-43 in astrocytes can lead to their activation, which gets abrogated when PKR is silenced, indicating TDP-43 modulation of PKR activity [61]. Perhaps, TDP-43 accumulation status can be relayed through the activation of PKR, increasing eIF2α phosphorylation. Furthermore, FUS is found to mediate ER stress by interacting with protein disulfide isomerases [62]. Interestingly, the pathology of mutant FUS does not involve eIF2α phosphorylation, though translation is still impaired, possibly via RNP-associated entrapment of required components [63].

An interesting finding in chick embryo neural cells suggested that *C9orf72*-associated DPR translation was dependent on a unique initiation site (CUG) and eIF2A function. Furthermore, the expression of the DPR instigated the ISR, causing phosphorylation of eIF2α and attenuating cap-dependent global translation but with continued DPR expression (via RAN translation) [64]. In addition, similar results were observed in another ALS model system wherein non-canonical translation initiation (RAN translation) was found to be sensitive to eIF2D functions [65]. The pathology of FTD/C9orf72 DPRs could involve ER stress and PERK-mediated phosphorylation of eIF2α in specific brain regions [66]. A recent investigation of SOD1 mutant-associated ALS reported the phosphorylation of eIF2α to be linked with mutant protein expression. Interestingly, this phosphorylation occurred even in the absence of its known ISR-dependent kinases. Moreover, mutant protein accumulations can be sensed by HSP90, leading to the activation of PKCδ, which in turn phosphorylates MARK2. Activated MARK2 can be implicated as a new proteotoxicity-responsive kinase for eIF2α phosphorylation in ALS pathology [67]. These findings contribute to an understanding of the critical mechanisms that inhibit global protein synthesis as a part of the cellular response to proteotoxicity caused by different misfolded protein pathologies in ALS. 

### 2.5. Misfolded Proteins Lead to Mitochondrial Dysfunction

The increasing accumulation of mutant misfolded toxic protein species can mediate their transfer into the mitochondria, making its dysfunction one of the pathological hallmarks of several protein aggregation-based disorders (Figure 1E). In ALS, mutant C9orf72, TDP-43, and SOD1 associate with mitochondria [68,69,70]. The pathology of mitochondrial dysfunction can be characterized by impaired oxidative phosphorylation, ATP loss, the generation of ROS, a defective mitochondrial protein import mechanism, dysfunctional dynamics, altered calcium handling, and finally, the initiation of apoptotic signals [71]. The misfolded SOD1 accumulates inside mitochondria can potentially damage oxidative phosphorylation in the electron transport chain (ETC) [72]. This damage is linked with a sequestration of ETC proteins with misfolded aggregates that can potentially generate ROS. Furthermore, an accumulation of the toxic FUS protein in the cytosol allows the trapping and co-accumulation of ETC-related mRNA molecules, further attenuating energy production in the mitochondria [73]. C9orf72, on the other hand, is known to travel in the mitochondria to help the assembly of complex I in ETC. The ALS-associated decreased function of the C9orf72 protein can hamper complex I activity, ultimately generating ROS [74,75]. TDP-43 can aberrantly accumulate inside and on the outer surface of mitochondria, where it may block the proteasome mechanism and forfeit mitochondria-associated proteostasis [69,74,76]. This aberrant localization of mutant TDP-43 also promotes complex I disassembly via its binding to its subunit mRNAs [77]. Furthermore, an aggregation of SOD1 in the intermembranous space of mitochondria can block the outer membrane’s VDAC for calcium transport [78]. Importantly, the generation of ROS can potentially increase free calcium ions and activate mitochondrial permeability transition pore (mPTP), initiating the leakage of mitochondrial material, such as calcium, mitochondrial DNA, and finally, cytochrome c, for activating apoptosis [79,80]. The mitochondrial dysfunction associated with ALS-linked mutants may form a critical part of ALS pathogenesis.

### 2.6. Damage-Inducing Aberrant Immune Cells

The pathogenesis of the disease is also postulated to be associated with the altered activation of immune cells that can subsequently release toxic factors responsible for motor neuron damage (Figure 1F). The activation of NF-κB/AP1/STING can potentially promote innate immune signaling and the transcription of several immunomodulatory molecules. Studies have found that under in vitro and in vivo conditions, mutant SOD1 in astrocytes, mast cells, macrophages, and microglia, can potentially activate NF-κB signaling. Further, attenuating this signaling can serve as a therapeutic measure for ALS conditions [81,82,83]. Another study shows that the TDP-43 wild-type protein may compete with the NLS of p65 for nuclear transportation, thus reducing NF-κB activity [84]. The TDP-43 mutant forms present extracellularly can associate with microglial CD14 surface molecule, resulting in its activation. The activated CD14 receptor can promote an inflammatory response via the instigation of NF-κB, AP1, and NLRP3 inflammasome [85]. Moreover, newer findings show that mutated TDP-43 can localize to mitochondria and may cause a cytosolic release of mtDNA. The mtDNA in cytosol can activate the cGAS/STING pathway, which can further initiate an inflammatory response [86]. The mutant FUS is found to mediate TNF-α-dependent neuronal toxicity. Increased accumulation of mutant FUS in astrocytes can lead to their activation and a subsequent release of TNF-α in the conditioning medium for motor neurons. Furthermore, the toxic effects of TNF-α could be retarded by inhibiting NF-κB activity in astrocytes [87]. Similarly, in C9orf72 pathology, immune cells are activated and demonstrate enhanced levels of pro-inflammatory molecules [88]. However, the underlying immunologically related signaling is less clear in the case of C9orf72-mediated toxicity [82].

### 2.7. Glutamate Toxicity

Astrocytes are also responsible for critically regulating the concentration of glutamate in the synaptic gap. An excessive concentration of glutamate in the synapse is found to be responsible for generating excitotoxicity. ALS involves an enhanced release of the (excitatory) glutamate neurotransmitter [89] (Figure 1G). The activation of glutamate signaling is antagonized with the help of GABAergic neurons that potentially inhibit the excitatory functions of glutamatergic neurons. In addition to this, astrocytes can potentially regulate the glutamate concentration in the synapse by enhancing the reuptake of glutamate through the GLT-1 (EAAT2) transporter. ALS-associated mutant proteins can impair all the above mechanisms that regulate glutamate-mediated neurotransmission, generating excitotoxicity. For instance, TDP-43 was found to be necessary for the pre-processing of the mRNA of *GAD1* (which is essential for GABA synthesis) and, thus, for maintaining the level of GABA in the *Drosophila* nervous system [90].

Furthermore, TDP-43 is also responsible for reducing the highly sensitive glutamate receptors GLT-1 and GLAST from the astrocyte membrane [91]. Therefore, the generation of mutant TDP-43 that accumulates in the cytosol can potentially enhance hyper-excitability and axonal transport defects, increasing toxicity in ALS [92,93]. The reduced sensitivity of astrocytes to glutamate is also eminent in SOD1G93A pathologies, and the pathway follows the reduction in the GLT-1 and GluR1 (glutamate receptor) levels [94]. Moreover, the aggregates of mutant SOD1 in astrocytes can potentially enhance AEG1, which is responsible for the nuclear localization of YY1, an inhibitor of EAAT2 expression [95,96]. Mutant SOD1 was also found to enhance the efflux of glutamate in ALS by increasing the levels of intracellular calcium ions that perhaps activate calmodulin-based kinase II to enhance synapsin I phosphorylation, helping glutamate vesicle membrane fusion [97]. Similarly, FUS mutants are found to participate more in synaptosomal localization along with having abnormalities in the morphologies of synapses, leading to reduced GABAergic and abnormal glutamatergic neuronal activity [98]. The C9orf72 DPR can instigate an accumulation of excess glutamate extracellularly and excess calcium intracellularly in a *Drosophila* disease model [99]. In the case of astrocytes, DPRs can potentially hamper the process of glutamate reuptake [100]. Models with expanded *C9orf72* possibly exhibit de-regulated GABA release, which, combined with the above mechanisms, can lead to excitotoxic damage to neurons [101].

### 2.8. Loss of Proteostasis

ALS is characterized by mutant protein aggregates that arise during protein conformational disorders. The development of TDP-43 accumulates often damages UPS (Figure 1H). Aberrant TDP-43 can undergo ubiquitination and associate with proteasomal chaperone proteins such as PSMG2 and PSD13, leading to proteasomal dysfunction [102]. The accumulated mutant TDP-43 and FUS are reported to undergo ubiquitination and disturb the cellular balance of free ubiquitin due to their sequestering in the protein aggregates [103]. Moreover, TDP-25, consisting of the TDP-43 aggregation-prone prion-like domain, is sequestered with stalled proteasome subunits that are unable to undergo the conformational changes necessary for enzymatic activity [104]. Interestingly, TDP-25 can impair chaperone-assisted autophagy, though it was found that the BAG1 co-chaperone is responsible for targeting TDP-25 towards the proteasome, whereas upon overexpression, BAG3 may be involved in targeting the mutant TDP-25 towards autophagy degradation [105]. Though SOD1 mutants are well known to decrease proteasomal enzymatic functions, they can also be responsible for the dysfunction in the autophagy pathway [106,107].

DPRs can potentially aggregate in both p62 (autophagy receptor)-positive and -negative ubiquitinated forms. This can possibly lead to an increase in substrates for the UPS, leading to a greater load and probably causing their impairment [108]. In contrast, TDP-43 aggregation may increase the likelihood of autophagy initiation through upregulated TFEB translocation and reduced dynactin 1, leading to the impaired fusion of the autophagosome with the lysosome [109]. A similar effect can be seen during FUS accumulation that can potentially block macroautophagy in a Rab1-dependent manner [110]. Finally, chaperones that are primarily involved in dealing with ALS-linked mutant protein aggregates can themselves become sequestered in such accumulates. Consequently, several protein folders and chaperones are known to be deficient in function during the initial stages of disease pathogenesis [111]. Several chaperones such as HSP70, HSC70, and VCP, co-chaperones such as BAG3, HSP40 (DNAJ family), CHIP, and calreticulin and protein folders such as HSP110 (HSPH family), proSAAS, and MIF are reported to be enriched in SOD1, C9orf72, FUS, and TDP-43 aggregate protein compartments [112,113,114,115,116]. It is, therefore, clear that chaperones are the primary interactors of mutant proteins, and their deficient functions can aggravate the accumulation of misfolded proteins in ALS. This makes chaperones an interesting target for developing therapeutic strategies against ALS-based mutant proteins, and in the upcoming sections, we will discuss the updates and key studies carried out in this respect.

## 3. Employing Neuroprotective HSP70 in Delaying ALS-Associated Toxicity

**Figure 2:**
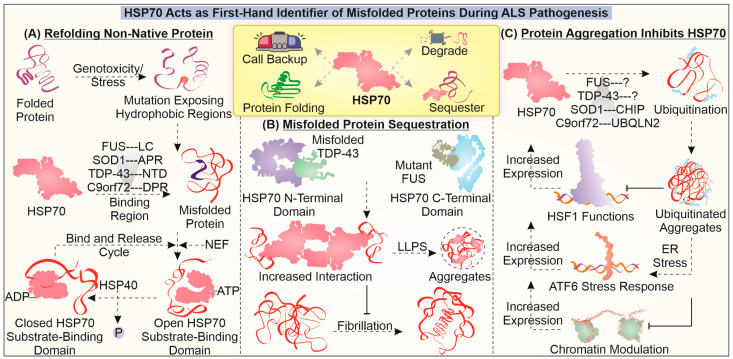
**Role of HSP70 in dealing with mutant proteins in ALS:** The figure illustrates the fundamental involvement of HSP70 in dealing with misfolded aggregates and their associated toxicities during the pathogenesis of ALS. HSP70 is found to engage in specifically identifying aberrant protein conformations of ALS-associated mutant protein and enhance their refolding (**A**), initiate sequestration (**B**), and instigate their clearance. The aggregation of misfolded proteins can entrap HSP70 and HSF1, reducing HSP70 levels during ALS (**C**).

The development of mutant protein aggregates and inclusions during the pathogenesis of ALS can primarily be identified and dealt with via the help of cellular protein chaperone systems. Among various cellular chaperones, HSP70 (HSPA) acts as the major primordial yet essential molecule involved in sensing the presence of aberrant protein species (Figure 2). HSP70, as the name suggests, is a 70 kDa chaperone that utilizes ATP to form an association with its substrates and may be responsible for their folding/refolding, disaggregation, and degradation [117]. Different isoforms of HSP70 (Grp78, HSC70, and HSP70) are present, utilizing similar mechanisms that may be associated with different co-chaperones or other molecules to complete the above-mentioned functions [118].

In TDP-43 pathology, HSP70 interacts with mutant TDP-43 and initiates its LLPS. Additionally, the presence of HSP70 in TDP-43-positive inclusions can help maintain its liquid nature by avoiding the transition to amyloid aggregate formation [119,120]. A similar liquid phase transition tendency of HSP70 was noted in the case of FUS pathology, wherein its occurrence in FUS-related stress granules was associated with reduced liquid-to-solid (amyloid-like) transition. It is interesting that HSP70 interacts with mutant FUS with the help of its C-terminal domain and that it may interact with TDP-43 using its N-terminal domain [116]. In the case of mutant SOD1, HSP70 can strongly interact with aggregation-prone regions [121]. HSP70 is also believed to interact with DPRs found in the C9orf72 model and to potentially reduce the toxicity mediated due to their aggregation [122]. These critical reports present a description of HSP70 involvement as a first-hand identifier and interactor of misfolded proteins. The interaction with HSP70 is believed to be critical in maintaining the liquid nature of accumulates and avoiding the generation of amyloid-like properties.

The interaction between aberrant proteins and HSP70 can also lead to their degradation with the help of the proteasome pathway or inside the lysosome. For instance, HSP70 may enhance the clearance of mutant SOD1 by interacting with both CHIP E3 ubiquitin ligase and mutant SOD1, delivering it to the proteasome [123]. HSP70 has also been reported to be associated with DPRs as a client, while also interacting with UBQLN2, a shuttle protein that links the HSP70–DPR to proteasome assemblies, enhancing the degradation of DPRs [120]. HSP70 can potentially restrict the development of toxic degradation-resistant misfolded forms of proteins. Additionally, the induction of HSP70 along with that of other components of the PQC system can provide protective effects to cells afflicted by a ALS-specific mutant protein, possibly via enhanced degradation [124,125]. Another important observation that is seen during the pathogenesis of the disease is the entrapment of HSP70 and its reduced functioning. The loss of HSP70 function during the pathogenesis could play two major toxic roles; one is the generation of solid misfolded protein-containing accumulates, and another is the restriction of other stress-responsive functions of HSP70. 

Furthermore, in a study, in which motor neurons were subjected to heat stress with mutant SOD1 expression, there was no increase in HSP70 levels. One of the reasons could be the impairment associated with HSF1 (a transcription factor for genes encoding chaperones) activity, possibly leading to a high threshold requirement for generating a stress response in these neurons [126]. The entrapment of HSP70 in protein aggregates can hamper its ability to instigate stress response pathways via making it functionally unavailable in motor neurons [127]. Can this factor also contribute to the above-mentioned high threshold in motor neurons? Results observed in another study in which SOD1G93A mutant expression was observed indicated an enhancement of the heat shock response and HSP70 expression upon induction by histamine [128]. In the case of glial cells with SOD1G93A expression, their activation and inflammatory NF-κB functions were increased in the response to stress, possibly due to reduced HSP70-related HSR [129]. Interestingly, the use of HDAC inhibitors can alter the histone acetylation that could be responsible for enhanced expression of HSPs. The possible defects associated with the activation of HSP70 in mutant FUS protein-related ALS could not be reversed even upon using HDAC inhibitors [130]. Such data imply that the expression of HSP70 is masked in presence of ALS-associated mutant proteins, which can limit its ability to instigate the stress-responsive pathways.

In addition to the above-mentioned reports, the critical involvement of HSP70 in reducing misfolded protein-based toxicity in ALS can be gauged from its potential to ameliorate pathological hallmarks of the disease. For instance, as discussed earlier, the expression of ALS-related mutant proteins is associated with faulty DNA repair pathways, leading to greater DNA damage. Overexpression of HSP70 during UV irradiation can be responsible for protection against DNA damage to a certain extent [131]. Furthermore, HSP70 is reported to possess RNA-binding properties, wherein it supposedly binds with AU-rich regions of RNA, requiring at least 30 nucleotides for binding [132]. This RNA-binding property of HSP70 can potentially modulate the(stress-responsive) protein expression profile by regulating mRNA decay. Moreover, HSP70 is critical to the ribosome-associated folding of nascent polypeptides, thus helping in protein synthesis [133]. Mitochondrial fragmentation is associated with protein aggregation, and HSP70 can promote SIRT3-mediated inhibition of mitochondrial fission. Moreover, HSP70-dependent SIRT3 upregulation can help to maintain a balance between mitochondrial fission and fusion mechanisms, thereby inhibiting the activation of apoptosis [134]. The enhancement of mitochondrial functionality by HSP70 is also attributed to its ability to reduce ROS production [135]. 

Exogenous treatment with the HSP70 protein has been described to protect against neuroinflammation-associated damages in protein misfolding-based Parkinson’s disease, indicating a possible protective role against ALS-associated neuroinflammation too [136]. Another study carried out in the SOD1G93A mouse model demonstrated that intraperitoneal administration of HSP70 leads to a delay in the onset of symptoms along with an improvement in survival [137]. Such findings imply the specific potential of HSP70 in dealing with ALS-associated cellular and molecular anomalies. Perhaps, targeting HSP70 expression could potentially ameliorate the generation of toxic protein inclusions and instigate several response mechanisms such as the UPR, HSR, and DNA damage response as its auxiliary functions by relaying the cellular damage status. 

## 4. Anti-Apoptotic HSP27 Provides Multifaceted Protection against Proteotoxicity

**Figure 3:**
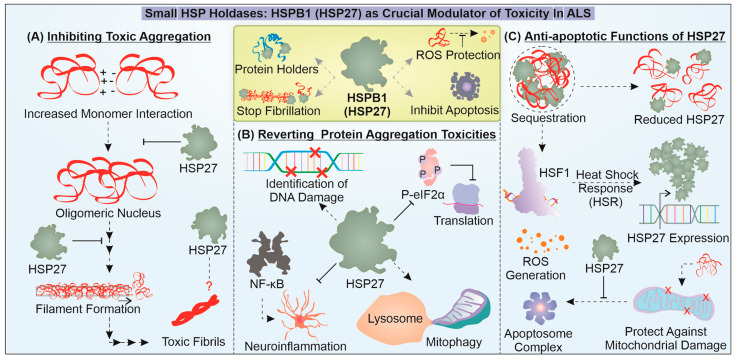
**Unveiling the crucial roles of HSPB1 (HSP27) in combatting mutant proteins linked to ALS.** The figure illustrates how HSP27 is critical in mitigating toxicity-associated effects of misfolded aggregates during the progression of ALS. HSP27 is found to inhibit the toxic amyloidosis of mutant aggregation-prone proteins during ALS (**A**) and enhance the cytoprotective response against protein aggregation-associated toxicities (**B**). In addition, it can also act as an anti-apoptotic protein (**C**).

The cellular chaperone system also consists of a class of small chaperones that perform a vital role in maintaining proteostasis. HSPB1, also known as HSP27, belongs to the small HSP family, which consists of an α-crystallin domain (ACD) and functions by forming large oligomers of more than 30 monomers, with a mass of around 800 kDa in such oligomeric states [138,139]. They function essentially as holdases and, thus, do not require ATP hydrolysis for mitigating protein conformation abnormalities, unlike HSP70 (Figure 3). However, HSP27 and HSP70 are essential for refolding misfolded proteins or initiating their removal. The HSPB1 phosphorylation status seems to be a primary regulator of its activity. It is known for its potential involvement in tumor conditions, ability to augment the activation of apoptosis, and response to cellular stress-generating proteotoxicity [140,141]. 

In a recent report, HSPB1 was found to interact with low-complexity (LC) TDP-43regions during different proteotoxic stress responses and aid in the disassembly of TDP-43 droplets back into a liquid-like state. Essentially, depleting HSPB1 led to the conversion of TDP-43 into a toxic degradation-resistant accumulation. Additionally, a reduction in HSPB1 was noted in spinal cord motor neuronal cells with TDP-43 pathology [142]. Another interesting report demonstrates the diverse function of HSPB1 in the case of FUS pathology. HSPB1 is found to interact with FUS and inhibit its LLPS and aggregation as well as its targeting of stress granules. The mechanics of this activity are rooted in the weak interaction of HSP27 with the LC regions of FUS and possibly attenuate the long-range inter/intra molecular interaction of FUS-LC [143]. C9orf72 DPR expression is found to activate HSF1 and subsequent HSR activation includes HSPB1 upregulation as a response to DPR-dependent toxicity [144]. It is still unclear what role HSPB1 plays in mitigating DPR aggregation potential. Furthermore, HSPB1 is reported to inhibit the growth of SOD1 aggregates but does not reduce the formation of nuclei in vitro, suggesting that they associate with mutant SOD1 oligomers at a particular stage of aggregation [145]. These studies indicate the specific potential of HSP27 to regulate the growth of misfolded protein oligomers. Additionally, it can also restrict the conversion of non-toxic states of misfolded protein into toxic degradation-resistant states.

Apart from providing neuroprotection through direct interaction with misfolded mutant proteins, HSP27 is well known to mediate anti-apoptotic effects. The combined expression of mutant SOD1 and HSPB1 in the ALS model of transgenic mice showed a delay in the appearance of symptoms and improved motor neuron function [146]. However, in another study, when only the HSPB1 expression construct was introduced in cells expressing mutant SOD1, no cytoprotection was observed [147]. Moreover, HSP27 is present in decreased levels in motor neurons with an ALS-related mutant protein, and this reduction is often found to be associated with increased neuronal loss [148]. Furthermore, an interesting study demonstrated that a PEP-1-HSP27 fusion protein, expressed in a bacterial system and transduced in cells expressing mutant SOD1, could limit protein aggregation and ROS-mediated damages [149]. However, when mice with SOD1G93A were crossed with mice with a ubiquitous expression of HSPB1, the transgenic (offspring) mice did not exhibit any delay in the onset of the disease or improvement in survival [150]. In TDP-43 pathology, cellular stress caused by ROS, the inhibition of proteasome or a loss of function of HSP70 prompts HSPB1 function and sequesters TDP-43 to reduce toxicity [140]. This implies that HSPB1 probably functions in accordance with HSP70 and in response to damages caused due to a toxic accumulation of misfolded mutant proteins. Thus, HSPB1 may enter the disaggregation program at a later stage and could work to circumvent the toxicities caused by protein aggregation.

Furthermore, to elucidate the cytoprotective involvement of HSPB1, inspiration can be drawn from tumor conditions in which it is found to be upregulated. The protein interaction analysis carried out for prostate cancer cells revealed that HSPB1 was associated with 226 interactors, including DNA repair proteins, and could potentially help in the recognition of DNA damages [151,152,153]. Under misfolded protein stress, activation of HSPB1 can potentiate its binding to promoters that may or may not contain a TATA site, possibly with the help of the SP1 transcription factor, and initiate the expression of several cytoprotective genes [154]. Additionally, HSPB1 levels may not necessarily avoid the synthesis of polypeptides under cellular stress involving a misfolded protein but may enhance the recovery of translation by increasing the dephosphorylation of eIF2α [155].

During aging in cardiac cells, improving HSPB1 expression can help reduce aging-associated defects. This activity of HSPB1 is linked with the enhanced removal of dysfunctional mitochondria through mitophagy and reduced oxidative damage [156]. In addition to these functions, HSPB1 can specifically reduce cytokine formation by attenuating the inducement of NF-κB [157]. Even in the autonomous non-cell mechanism of ALS involving neuroinflammation, glial cells are reported to have upregulated HSPB1 in SOD1 pathology. Specific upregulation of HSPB1 in astrocytes can ameliorate astrocyte-dependent toxicity in SOD1-ALS [158]. Such reports place HSPB1 among the crucial modulators of the several damage control pathways during ALS pathogenesis. However, as mentioned earlier, under mutant protein stress, the exogenous expression of HSPB1 failed to provide cellular protection. However, when mild heat stress was given, protection was facilitated against the mutant ALS-linked protein, even upon HSPB1 depletion, possibly with the aid of other chaperones [147]. Designing a strategy to rescue different chaperones’ gene expressions and protein levels can help attenuate the development of new amyloid forms of the toxic protein. There is still uncertainty regarding the extent of protection offered by HSPB1 against proteotoxic stress, necessitating further research.

## 5. DNAJ Family Chaperones Aid in Solubilization and Clearance of ALS-Linked Pathogenic Variants

**Figure 4:**
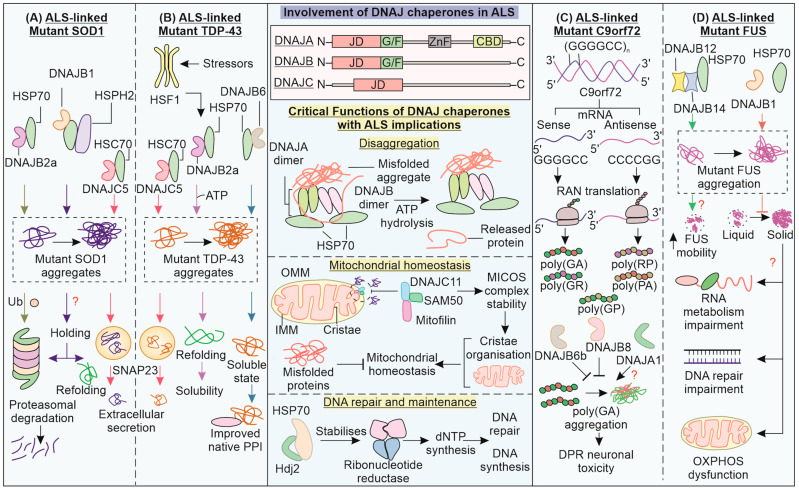
**Exploring the involvement of DNAJ chaperones in the ALS pathomechanism:** DNAJ proteins affect ALS pathomechanisms by influencing the folding status and solubility of SOD1, TDP-43, C9orf72, and FUS. (**A**) DNAJB2/B1 mediates clearance of mutant SOD1, possibly in a proteasomal-dependent manner, whereas DNAJC5 can mediate extracellular secretion of mutants TDP-43 and SOD1. (**B**) DNAJB2a, under the control of HSF1 and DNAJB6, can improve the solubility of mutant TDP-43. (**C**) C9orf72 DPRs are synthesized via the RAN translation of expanded *C9orf72* mRNA of both types. DNAJB6b and DNAJB8 increase the clearance of C9orf72 DPR accumulation, while the role of DNAJA1 is not clear. (**D**) DNAJB12 and DNAJB14, along with HSP70, inhibit the impaired phase separation of FUS, which could have implications for the mentioned cellular processes. Other critical functions of DNAJ proteins, such as disaggregation, mitochondrial homeostasis, and DNA synthesis and repair can have implications for ALS.

DNAJ (HSP40) chaperones have a characteristic J domain for interaction with HSP70 and promote its hydrolysis of ATP. ATP hydrolysis increases the HSP70 substrate protein interaction to aid folding, with nucleotide exchange factors (NEFs) restoring the ATP state of HSP70, resulting in a release of the folded substrate. DNAJ proteins are classified based on the domain types present—DNAJA, DNAJB, and DNAJC [159] (Figure 4). The J domain is present in the N-terminal, glycine/phenylalanine-rich (G/F) region, a Zn finger motif, and a C-terminal-binding domain in DNAJA proteins. DNAJB proteins have a J-domain and G/F region, while DNAJC only has a J domain [159].

Among DNAJ family chaperones, ALS-linked variants of DNAJC7 have been reported in a whole exome sequencing study on ALS patients of European origin [160]. However, clarity has yet to emerge in terms of DNAJC7 pathogenic variants and their ALS pathomechanisms, given its rare incidence (Chinese, Taiwanese, and Japanese ALS cohort screening) [161,162,163]. DNAJB2 (Hsj1a) is reported to reduce the aggregation of SOD1G93A in a J domain- and ubiquitination-dependent manner in an ALS mouse model [164]. Increasing levels of DNAJB2 can improve motor unit function as well as motor neuron survival. DNAJB1 and DNAJB7, along with HSP70 and HSP110 (NEF), can reduce mutant SOD1 (A4V) aggregation [118]. Furthermore, HSP40, in combination with HSP70, can remove intracytoplasmic aggregates of mutant SOD1, leading to improvements in cell viability and neurite outgrowth [165]. 

Neuron-specific DNAJB2a aids in refolding and solubilizing mutant TDP-43 with the help of its J domain, handing it over to HSP70 [166]. The reduction in insoluble and hyper-phosphorylated fractions of mutant TDP-43 is brought upon by refolding. In ALS patients, reduced HSP40 and HSP70 chaperone levels can contribute to TDP-43 proteinopathy and consequent cell death. Another HSP40, Sis1 (a DNAJB1 homolog in yeast) can alleviate TDP-43 aggregate toxicity in cells, including the inhibition of proteasomal machinery [167]. DNAJB6 possibly interacts with the TDP-43 C-terminal domain, keeping it in a soluble state and preventing its aggregation [168]. Chaperone availability can be critical in preventing TDP-43 self-aggregation via its C-terminal domain. Recently, misfolding-associated protein secretion (MAPS) has been reported, where misfolded cytosolic proteins are secreted out of the cell via the endocytic pathway, aided by the chaperone activity of ER membrane-linked deubiquitinase USP19 and DNAJC5 in an autophagy-independent manner [169,170]. 

DNAJC5, combined with HSC70, helps the extracellular secretion of neurodegeneration-associated misfolded proteins, such as SOD1, TDP-43, and tau, possibly upon increased proteotoxic burden [171,172]. While this may serve as a cytoprotective measure upon the overwhelming of the refolding/degradative machinery, an excessive release of such pathogenic proteins can become a transmission route for neighboring cells [173]. TDP-43 is also known to aid in localizing CHCHD10 (a mitochondrial intermembrane space protein) to the nucleus, while CHCHD10 prevents the cytosolic targeting of TDP-43 [174]. CHCHD10 depletion is further associated with the mitochondrial localization of TDP-43, where it can promote mitochondrial dysfunction. In motor neurons, ER stress can be caused by mutant SOD1, which is found to be responsible for activating the apoptotic mechanism [175]. A post mortem investigation of an ALS-related spinal cord revealed the upregulation of DNAJB9 and DNAJC10 due to ER stress [176]. These co-chaperones, along with BiP (HSP70), aid in the removal of aberrant protein via ER-associated proteasomal degradation (ERAD) [177]. Maintaining ER homeostasis is another crucial aspect of the DNAJ chaperone’s role in cytoprotection.

C9orf72 is a guanine NEF protein that can exhibit hexanucleotide repeat expansion (GGGGCC) in its gene’s intronic region. In an interactome-investigative study, DNAJB6b and DNAJB8 were reported to reduce the aggregation of poly(GA) DPRs [112]. These chaperones are also known to reduce the aggregation of other neurodegenerative disorder-associated proteins (polyQ and α-synuclein) [178,179]. Such studies indicate the expression of DNAJB6/8 in improving clearance of neurodegeneration-associated proteins, though DNAJA1 is also reported to localize with DPRs. Furthermore, autopsy studies from different brain areas reflected the involvement of BAG3, VCP, and its adaptor UBXN in poly(GA) accumulates. The functions of C9orf72 require its interaction with SMCR8, forming a complex that affects vesicle transport. SMCR8 is known to be reduced in ALS pathology and can potentially interact with chaperones of the DNAJ family—DNAJA1, DNAJA3, and DNAJC7 [180,181]. It will be interesting to understand any potential association with or modulation of C9orf72 by these chaperones. 

ALS-related FUS variants can lead to the solidification or fibrillation of the core of their liquid phase inclusions, involving stronger protein–protein interactions and decreased diffusion and dynamicity [182]. FUS LLPS is also required for recruiting and retaining proteins of the DNA damage response (DDR) at DNA damage sites, such as Ku80 and SFPQ [183]. An interesting effect of this abnormal phase transition could be impaired DNA repair via the reduced ability of molecules involved in DNA repair to diffuse. The earlier-mentioned LC region towards N-terminal and the RGG-rich region towards the C-terminal of FUS can contribute to its aggregation [184,185]. The full-length isoform DNAJB14-FL, along with DNAJB12 and HSP70, prevents mutant FUS aggregation while also increasing its mobility [115]. The G/F region of DNAJ proteins can contribute to their capability of phase transition into cytoplasmic SGs under stress conditions, and mutations in this region disturb proper phase separation and DNAJB1 (Hdj1)–FUS co-localization [186]. DNAJB1 can stabilize the liquid phase of FUS, preventing its aggregation. Overexpression of DNAJ chaperones has already been reported to alleviate neurodegeneration protein-associated aggregation and toxicity, including ALS-linked FUS and TDP-43 [167,187]. 

Recently, DNAJA and DNAJB homodimers have been shown to form a complex with two HSP70 proteins, which can generate entropic pulling forces for disaggregation [188]. Another HSP40, DNAJC11 has been identified as a part of the MICOS complex along with SAM50 and mitofilin [189]. The splicing mutant of DNAJC11 is associated with abnormalities in mitochondrial crista organization and ventral horn motor nerve cell pathology in a mouse model, similarly to ALS. Deviation in the levels of DNAJA3, a mitochondrial DNAJ chaperone, leads to mitochondrial fragmentation in a Drp1-based manner [190]. DNAJA3 is also responsible for the phosphorylation of the p65 subunit of NF-κB via the stabilization of the IKKβ/IκB/p65 complex [191]. HDJ2 also interacts with and stabilizes ribonucleotide reductase subunit R2B, which is involved in DNA synthesis and repair via the synthesis of new dNTPs [192]. Interestingly, the mitochondrial HSP40/70 machinery is also essential for mitochondrial DNA maintenance and replication, while a depletion of these chaperones leads to DNA loss [193]. Apart from being directly involved in proteostasis, the involvement of DNAJ chaperones in mitochondrial homeostasis (morphology, dynamics, and DNA replication) as well as DNA repair, makes them significant players in ALS pathology.

## 6. Small Chaperone HSPB8 Provides Cytoprotection by Promoting Autophagic Clearance of ALS-Linked Pathogenic Variants

**Figure 5:**
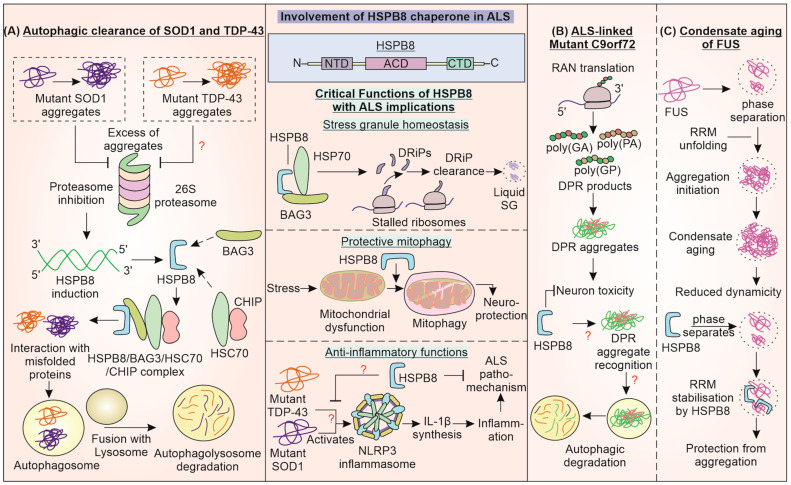
**Involvement of HSPB8 Chaperone in Pathogenesis of ALS:** The small HSP HSPB8 directs mutant SOD1, TDP-43, and C9orf72 proteins towards autophagic clearance upon proteasome inhibition. (**A**) HSPB8 performs this function, possibly in a complex with BAG3, HSC70, and CHIP. (**B**) HSPB8 promotes the autophagic degradation of C9orf72 DPRs. (**C**) HSPB8 also inhibits the condensate aging of the ALS-linked FUS protein, thereby preventing the hardening of stress granules. The critical functions of HSPB8 in stress granule homeostasis, mitophagy, and inflammation, can have implications for ALS.

HSPB8 or HSP22 is a small heat shock protein (sHSP) with a monomeric mass of around 21.6 kDa. It consists of a characteristic ACD between N- and C-terminal regions [194]. HSPB8 is known to have both chaperoning and autokinase activities. Functionally, it is involved in vesicle fusion and synaptic transmission, apoptotic regulation, and oxidative damage [195]. It has been reported to be involved in malfolded protein refolding, preventing aggregation, and directing them to degradation systems (Figure 5). Interestingly, an enhanced expression of HSPB8 is observed in anterior horn motor neurons, which are known to survive up until the end stage of ALS [196]. Ganassi et al. have demonstrated HSPB8 as a part of a protein complex consisting of chaperone HSP70 and co-chaperone BAG3 in the surveillance machinery for stress granules [197]. It maintains the homeostasis of SGs (granulostasis) by aiding the degradation of polypeptides formed from premature translation termination known as defective ribosomal products (DRiPs), keeping SGs in a liquid-like state.

Dysfunction of this machinery can hamper the degradation of DRiPs, leading to solid-like behavior or aggregation of SGs. The consequently decreased dynamicity and disassembly can result in a failure to restore protein translation. HSPB8 in SGs maintains DRiPs in a folding state that can be acted upon by BAG3 and HSP70 [197]. Moreover, HSPB8, along with BAG3, targets ubiquitinated misfolded proteins and p62 towards the juxtanuclear aggresome upon proteasome inhibition [198]. HSPB8 is also reported to promote neuroprotective mitophagy during ischemia/reperfusion damage in mice, maintaining mitochondrial function and preventing neuronal apoptosis [199]. Recently, lentiviral delivery of HSPB8 to mouse brains alleviated cerebral injury via maintaining levels of proteins of tight junctions and promoting autophagy, thus preserving blood–brain barrier (BBB) integrity [200]. HSPB8 also inhibits activation of NLRP3 and mitochondrial fission while improving cognition in diabetic mouse models. ALS-linked SOD1G93A and TDP-43 can activate the NLRP3 inflammasome in microglial cells [201,202]. Cumulatively, such reports indicate HSPB8 involvement in homeostasis via the regulation of cellular processes that are reportedly impaired in ALS. Like DNAJ chaperones, HSPB8 can also influence the aggregation of major pathogenic ALS-linked variants of SOD1, TDP-43, C9orf72, and FUS.

HSPB8 promotes the autophagic clearance of pathogenic aggregation-prone proteins in a BAG3 dependent manner [203]. This is possibly achieved with the aid of HSPB8 complex forms with BAG3, HSC70, and CHIP to increase the autophagy of aggregates of mutant SOD1, while the proteasome function of the cell is blocked by these aggregates [196]. This effect was studied in a SOD1G93A ALS mouse model and can provide an opportunity for therapeutic strategies for ALS. The BAG3 interaction with autophagy receptor protein p62 possibly contributes to the HSPB8-promoted autophagy of misfolded proteins [204]. Crippa et al. also exhibited HSPB8 facilitating autophagic degradation of accumulations of misfolded TDP-43 as well as TDP-25 [205]. The microtubule polymerization inhibiting drug colchicine and DNA intercalator doxorubicin have also been shown to increase the expression of HSPB8, aiding in misfolded protein clearance. Phase II of a randomized clinical trial for ALS treatment using oral colchicine tablets was completed in 2022 (NCT03693781) [206]. Recently, a high-throughput screening identified inducers of HSPB8 at both mRNA and protein levels that promote autophagic degradation of mutant misfolded protein SOD1 [207]. The levels of sHSPs, HSPB8 and HSPB5 are reported to increase in astrocytes in the spinal cord of ALS patients with short-duration disease, possibly aiding their response towards motor neuronal cell protection [208].

Interestingly, autophagic clearance of nucleotide repeat-expanded *C9orf72*-translated DPR products, except poly(PR) DPR peptides, was reported in immortalized motor neurons. Increasing HSPB8 expression enhanced the clearance of a majority of DPR products, possibly in an autophagy-dependent manner [209]. The pathways responsible for DPR product toxicity are unclear, though aggregate formation is involved. DPR products have recently been reported to sequester autophagy receptor p62, which is also an ALS-associated gene [210]. Similarly to DNAJB14 and 12, HSPB8 is also reported to regulate the phase transition of RNA-binding protein FUS. Recently, FUS was observed to undergo condensate aging from the liquid to solid phase upon the unfolding of its RNA recognition motif (RRM). HSPB8 prevents this fibrillation of FUS inclusions by stabilizing RRM through an interaction with its ACD [211]. The disease-associated mutant of HSPB8 at lysine residue 141 (in ACD) loses the protective function of preventing FUS condensate aging. The regulation of proteostasis of ALS-linked proteins by HSPB8 demonstrates that it is a critical molecule that can be further investigated for its core cellular functions and therapeutic potential in ALS.

## 7. Conclusions

The misfolding of mutant proteins responsible for ALS pathogenesis hampers cellular homeostasis, leading to an imbalance that generates toxic conditions. In addition to the blockage of cellular proteostasis, several stress-responsive mechanisms become deterred, causing a faulty transition of the cytoprotective response towards cytotoxicity. The generation of aberrantly folded non-native mutant proteins during the pathogenesis can be considered an intermediatory step, necessarily culminating in the activation of neuronal apoptosis. Further, the generation of misfolded proteins is regulated by crucial chaperone functioning, which again can become trapped or dysfunctional. Chaperones are actively involved in helping nascent or stress-generated non-native protein conformations to undergo a progressive folding process into native forms of protein. Key chaperones, such as HSP70, BAG3, and HSPB8, regularly patrol the cytoplasm to detect any protein molecules with non-native conformations. 

Upon detection of any of such toxic species, these chaperones may initiate an intrinsic refolding process or, if required, may lead to their clearance through either the proteasome or autophagy, as seen in case of HSP70, HSPB8, BAG3, DNAJB2, BAG1, and CHIP. Alternatively, for ALS-linked mutant proteins, such as FUS and TDP-43, chaperone proteins HSP70, HSPB1, HSPB8, DNAJB1, and DNAJA1 are actively involved in regulating the formation of LLPS-mediated membrane-less granules. In addition, chaperones such as DNAJC5 and HSC70 may also mediate the extracellular secretion of ALS misfolded proteins via an endocytic pathway to clear them from the cytoplasm. Small chaperones such as HSPB1, DNAJA, and DNAJB, along with HSP70 and HSP110, may critically initiate disaggregation, attempting to dissolve misfolded accumulates. A protein engineering strategy (a directed evolution involving a systematic selection of chaperone variants with improved functions) can be employed to enhance the activity and chaperoning potential of these critical disaggregates for the improved removal of mutant proteins [212,213].

Under excessive misfolding stress, aberrant protein accumulations may co-accumulate other intrinsically disordered proteins or chaperone interactors such as HSF1, leading to their entrapment and impairment. Such loss of HSF1 activity is found to further reduce the overall cellular chaperone capacity to deal with misfolded proteins. Moreover, reducing chaperones such as HSP70, HSPB1, DNAJB9, and DNAJC10 can further diminish their secondary functions, involving the activation of stress-responsive pathways. The stress response may include the activation of a heat shock response, ER stress-related pathways, DNA repair mechanisms, ISR, and mitochondrial stress responses. Additionally, HSP70, HSPB1, and HSPB8 may possess the ability to bind with a nucleotide or associate with nucleotide-binding proteins and, thus, regulate the expression of several genes. Moreover, recent investigations have found HSP70 to bind with specific mRNA transcripts and possibly regulate their metabolism or expression. Alternatively, HSP70 and HSPB1 may also activate transcription factors for stress-responsive genes. Such critical functions of chaperones represent their ability to interact physically with the misfolded protein and possibly relay their toxicity status at the genomic levels. 

**Figure 6:**
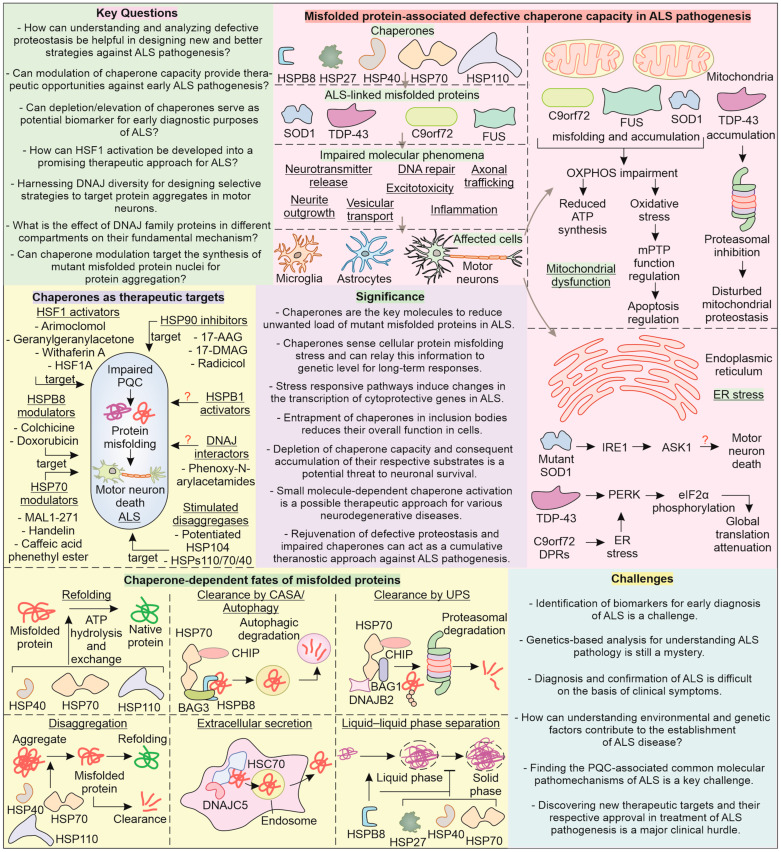
**Major key questions, challenges, and significance of chaperones in ALS pathogenesis:** The chaperone network in neuronal cells affects the folding, solubility, and aggregate formation status of ALS-related variants SOD1, TDP-43, C9orf72, and FUS. Their inability to perform these functions can result in impaired DNA repair, vesicular transport, excitotoxicity, axon branching, and trafficking. Mitochondrial dysfunction and ER stress are significant in ALS pathogenesis. Chaperone proteins should serve as candidates for therapeutic approaches for ALS to target the central pathomechanism of ALS, that is, impaired PQC and protein misfolding/aggregation. Chaperone proteins (HSP70, HSPB8, HSPB1, and DNAJ) as well as their transcription factor HSF1 can act as potential therapeutic targets. The modulators can increase the levels of chaperones, such as colchicine and doxorubicin, or directly interact with them, such as in the case of MAL1-271 and handelin. The chaperone action on misfolded proteins can induce their refolding, clearance via the UPS and autophagy, extracellular secretion, and disaggregation as well as regulate phase separation.

One of the significant hurdles in ALS therapy is the timely diagnosis of the condition before the appearance of clinical symptoms, after which it generally exhibits rapid progression. Currently, a major clinical challenge is the lack of curative treatment for ALS and the much less than desirable improvement in the survival period with approved drugs (Figure 6). A riluzole, edaravone, and taurursodiol and sodium phenylbutyrate combination (relyvrio) targets excitotoxicity, oxidative stress, mitochondrial health and HDAC, respectively, in ALS [214,215,216]. Sodium phenylbutyrate can also act as a chemical chaperone, with the ability to enhance cellular chaperone levels. Furthermore, CC100, a synthetic caffeic acid phenethyl ester (CAPE) that increases HSP70 levels has recently completed phase I clinical trials for ALS [217,218,219]. Augmenting HSP70 ATP hydrolysis can help its chaperone functioning, which can be targeted with the help of handelin and MAL1-271 and investigated for the possibility of therapeutic potential in ALS pathogenesis [220,221]. Furthermore, the functioning of HSP70 can also be targeted by inhibiting its co-chaperone, HSP40. Phenoxy-N-arylacetamide is reported to bind bacterial DnaJ (HSP40) and inhibit HSP70–HSP40 functioning. Moreover, phenoxy-N-arylacetamide can be used as a template to generate derivatives that may possess HSP40 activation properties [222]. 

The overall expression of chaperone functioning in cells is majorly attributed to the HSF1-mediated synthesis of HSPs. As discussed earlier, upon expression of ALS-linked misfolded proteins, the activation of the heat shock response (HSP synthesis) is attenuated, indicating HSF1 dysfunction. Therefore, it is intriguing to ask whether or not rescuing HSF1 expression can help ameliorate a misfolded pathology in ALS. Under normal conditions, HSP70 and HSP90 (HSPC family) bind to HSF1, preventing its activation, and hence, HSP90 inhibitors show potential to activate HSF1. In this respect, 17-AAG, a HSP90 inhibitor, can be investigated to improve chaperone-mediated clearance of ALS-linked proteins [223]. Indeed, the HSP90 inhibitor geldanamycin has been recently reported to promote degradation of DPRs, though in a proteasome-dependent manner [224]. However, inhibition of the HSP90 chaperone might overall reduce cellular chaperone capacity, and thus, further investigation is required to be conducted on the HSP90 inhibitor’s mechanism of action to limit the unintended effects [225]. Other compounds, such as celastrol, GGA, withaferin A, and HSF1A (activators of HSF1) can be investigated for improving cytoprotection in ALS pathogenesis [226,227,228,229,230,231]. The activation of HSF1 for targeting ALS anomalies through arimoclomol has recently completed phase III trials, reinforcing the significance of HSPs as a critical target in ALS [232]. 

The DNAJ class of chaperones perform critical modulation of toxic ALS-linked proteins in collaboration with HSP70. It is, therefore, significant to ask whether or not targeting the upregulation of J-domain proteins can help boost the cellular chaperone capacity. However, it is unclear whether targeting J-domain proteins can lead to any adverse impacts in ALS, given the diversity of their members and roles, necessitating still further investigation on their therapeutic potential. The protein aggregation pathway involves a critical accumulation of oligomers into nuclei that form a basis for the further toxic assembly of protein-generating degradation-resistant amyloid fibrils [233,234]. It is, therefore, significant to primarily target the accumulation of such misfolded protein oligomers’ conversion towards nuclei. During ALS pathogenesis, can upregulating key chaperones such HSP70, HSPB1, HSPB8, and DNAJ actively inhibit the generation of misfolded protein aggregation nuclei? Furthermore, targeting such misfolded protein aggregation intermediates can reduce the burden on the cellular proteolysis machinery. Drugs that target the upregulation of the proteasome or autophagy in combination with inducers of HSP70, HSPB1, HSPB8, and DNAJ can enhance the removal of ALS-associated mutant protein aggregates, potentially improving patient survival. Future research on the involvement of chaperones in cellular response to ALS-linked mutant proteins and in ALS pathogenesis can advance disease understanding, bringing us closer to more effective therapy. 

## Data Availability

Not applicable.

## Abbreviations

17-AAG,17-allylamino-17-demethoxy-geldanamycin; 17-DMAG, 17-Dimethylaminoethylamino-17-demethoxygeldanamycin; ACD, α-crystallin domain; AEG1, astrocyte-elevated gene 1; ALS, amyotrophic lateral sclerosis; AP-1, activator protein 1; ASK1, apoptosis signal-regulating kinase 1; ATM, ataxia telangiectasia-mutated; ATR, ATM and RAD3-related; BAG1/3, BAG family molecular chaperone regulator 1/3; BiP, binding protein; C306, cysteine residue 306; C9orf72, chromosome 9 open reading frame 72; CAMK, Ca^2^⁺/calmodulin-dependent protein kinase; CD14, cluster of differentiation 14 receptor; cGAS, cyclic GMP-AMP synthase; CHCHD10, coiled–coil–helix–coiled–coil–helix domain-containing protein 10; CHIP, carboxy terminus of Hsc70-interacting protein; Chk1/2, checkpoint kinase 1/2; CRM1, chromosomal maintenance 1; DPR, dipeptide repeat; DRiPs, defective ribosomal products; Drp1, dynamin-related protein 1; EAAT2, excitatory amino acid transporter 2; eIF2α, eukaryotic initiation factor 2 alpha; ERAD, endoplasmic reticulum-associated degradation; ETC, electron transport chain; FUS, fused in sarcoma; GABA, gamma-amino butyric acid; GAD1, glutamate decarboxylase 1; GCN2, general control nonderepressible factor 2 kinase; GGA, geranylgeranylacetone; GLAST, glutamate aspartate transporter 1; GLT-1, Glutamate transporter 1; GluR, Glutamate receptor; Grp78, glucose-regulated protein 78 kDa; HDAC, histone deacetylase; hnRNPA3, heterogeneous nuclear ribonucleoprotein A3; HRI, heme-regulated eIF2α kinase; Hsc70, heat shock cognate 71 kDa protein; HSF1, heat shock factor 1; HSP, heat shock protein; HSR, heat shock response; IKK, inhibitor of nuclear factor kappa-B kinase; IRE1, inositol-requiring enzyme 1; IκB, inhibitor of nuclear factor kappa B; MARK2, microtubule affinity-regulating kinase 2; MICOS, mitochondrial contact site- and cristaorganizing system; MIF, macrophage migration inhibitory factor; MMEJ, nicrohomology-mediated end joining; Mre11, Meiotic recombination 11; mtDNA, mitochondrial DNA; NF-κB, nuclear factor kappa B; NHEJ, non-homologous end joining; NLRP3, NLR family pyrin domain containing 3; NLS, nuclear localization signal; NPM1, nucleophosmin 1; NRE, nucleotide repeat expansion; NUPs, nucleoporins; OPTN, optineurin; OXPHOS, oxidative phosphorylation; PARP1, poly [ADP-ribose] polymerase 1; P-eIF2α, phosphorylated eukaryotic Initiation factor 2 alpha; PERK, PKR-like ER resident kinase; PKCδ, protein kinase C delta; PKR, protein kinase R; poly(GA), poly(glycine-alanine); poly(GP), poly(glycine-proline); poly(GR), poly(glycine-arginine); poly(PA), poly(proline-alanine); poly(PR), poly(proline-arginine); PP1, protein phosphatase 1; PPI, protein–protein interaction; PQC, protein quality control; PSD13, proteasome assembly chaperone 13; PSMG2, proteasome assembly chaperone 2; RAN, repeat-associated non-ATG; RBP, RNA-binding protein; RGG, Arg-Gly-Gly; ROS, reactive oxygen species; RRM, RNA recognition motif; SFPQ, splicing factor proline and glutamine-rich; SG, stress granule; sHsp, small heat shock protein; SMCR8, Smith Magenis syndrome chromosome region, candidate 8; SNAP23, synaptosomal-associated protein 23; SOD1, superoxide dismutase 1; STING, stimulator of interferon genes; TDP-43, TAR DNA-binding protein 43; TFEB, transcription factor EB; TNFα, tumor necrosis factor α; UBQLN2, ubiquilin 2; UBXN, UBX domain containing protein; UPR, unfolded protein response; UPS, ubiquitin proteasome system; USP19, ubiquitin-specific peptidase 19; VCP, valosin-containing protein; VDAC, voltage-dependent anion channel; WDR41, WD repeat domain 41; XRCC1, X-ray repair cross-complementing protein 1; YY1, Yin Yang protein 1.
